# Neural Basis of Self and Other Representation in Autism: An fMRI Study of Self-Face Recognition

**DOI:** 10.1371/journal.pone.0003526

**Published:** 2008-10-29

**Authors:** Lucina Q. Uddin, Mari S. Davies, Ashley A. Scott, Eran Zaidel, Susan Y. Bookheimer, Marco Iacoboni, Mirella Dapretto

**Affiliations:** 1 Department of Psychiatry, Stanford University School of Medicine, Stanford, California, United States of America; 2 Department of Psychology, University of California Los Angeles, Los Angeles, California, United States of America; 3 Ahmanson-Lovelace Brain Mapping Center, Semel Institute for Neuroscience and Human Behavior, Department of Psychiatry and Biobehavioral Sciences, David Geffen School of Medicine, University of California Los Angeles, Los Angeles, California, United States of America; Victoria University of Wellington, New Zealand

## Abstract

**Background:**

Autism is a developmental disorder characterized by decreased interest and engagement in social interactions and by enhanced self-focus. While previous theoretical approaches to understanding autism have emphasized social impairments and altered interpersonal interactions, there is a recent shift towards understanding the nature of the representation of the self in individuals with autism spectrum disorders (ASD). Still, the neural mechanisms subserving self-representations in ASD are relatively unexplored.

**Methodology/Principal Findings:**

We used event-related fMRI to investigate brain responsiveness to images of the subjects' own face and to faces of others. Children with ASD and typically developing (TD) children viewed randomly presented digital morphs between their own face and a gender-matched other face, and made “self/other” judgments. Both groups of children activated a right premotor/prefrontal system when identifying images containing a greater percentage of the self face. However, while TD children showed activation of this system during both self- and other-processing, children with ASD only recruited this system while viewing images containing mostly their own face.

**Conclusions/Significance:**

This functional dissociation between the representation of self versus others points to a potential neural substrate for the characteristic self-focus and decreased social understanding exhibited by these individuals, and suggests that individuals with ASD lack the shared neural representations for self and others that TD children and adults possess and may use to understand others.

## Introduction

Autism spectrum disorder (ASD) is a complex developmental condition in which fundamental social development and communication are compromised [1], [2], often with concomitant restricted interests and repetitive and stereotyped behaviors. The term “autism” is derived from the Greek word “autos”, meaning “self”. The first case studies of the condition include numerous descriptions of the solitary characteristics of the children examined. In describing one boy, Kanner (1943) writes “he got happiest when left alone, almost never cried to go with his mother, did not seem to notice his father's homecomings, and was indifferent to visiting relatives…He seems to be *self-satisfied*…almost to draw into his shell and *live within himself*...To get his attention almost requires one to break down a mental barrier between his inner consciousness and the outside world.” Kanner's early work includes numerous such references to the seeming indifference to social interaction and extreme self-focus exhibited by these children, who, as he observed, regarded contact with others as “interference”. In describing another child he writes that he “…behaved as if people as such did not matter or even exist” and describes one young girl as giving “the impression of being *self-absorbed*” [3]. While this and subsequent reports have highlighted the primary significance of altered self- and other-representations in ASD [4], [5], the question of what specific brain processes give rise to these phenomena remains open.

Despite recent efforts to use a cognitive neuroscientific approach to understand key components of the ASD symptomatology, there is still little consensus as to its precise neurobiological underpinnings. Several imaging studies have shown both structural and functional brain abnormalities in individuals with ASD (see [6] and [7] for reviews), but reports are still far from consistent. Impairments in the types of higher-order mentalizing essential to social cognition have long been implicated in autism [8], [9]. While previous work has focused largely on *interpersonal* cognition in autism, a recent shift has been towards understanding altered *intrapersonal* cognition in ASD. One recent study highlights broad impairments in both self-referential cognition and empathy as measured by a battery of scales designed to measure these constructs [10].

It has been suggested that the core symptoms of autism may result from a lack of the fundamental appreciation of the commonality between self and others [11]. Such interpersonal awareness may be mediated by a right fronto-parietal neural network [12]. Our previous work also suggests that in adults, a right fronto-parietal network responds to both self and other familiar faces, with greatest activity in response to stimuli most resembling the self-face [13], [14]. These regions engaged during self- and other-face processing overlap with areas that may contain mirror neurons [15], [16]. Recently, it has been proposed that mirror neuron dysfunction may underlie some of the symptoms characteristic of ASD, including deficits in social cognition [17]–[19]. Preliminary support for the mirror neuron dysfunction theory of autism comes from studies using fMRI [20], EEG [21], [22], transcranial magnetic stimulation (TMS) [23], EMG [24] and structural MRI [25] (see [18] and [26] for reviews). To date no neuroimaging study has attempted to examine whether such dysfunction contributes to aberrent self-other representations using facial stimuli in individuals with ASD.

Self-face recognition is evidenced by typically developing children around 2 years of age [27]. Many young children with autism exhibit a developmental delay in the acquisition of this ability, though the majority tested to date show evidence of some self-recognition [28], [29]. The ability to self-recognize is often thought of as indicative of an underlying self-concept [30], as it tends to develop in parallel with the use of personal pronouns (“I” and “me”) [31], and has only been reliably demonstrated in humans and great apes [32]. Little is currently known about the development of neural systems supporting self-recognition, and even less is known about the integrity of these systems in autism. The current study aimed to investigate the functioning of neural systems involved in shared representations for self and others in autistic children. We used a self-face recognition paradigm to test whether the autistic profile may involve abnormal functioning of fronto-parietal systems during self- and other-representation.

## Materials and Methods

### Participants

Eighteen high-functioning ASD and 12 age-and IQ matched TD (all male) children were recruited and compensated for their participation in this fMRI study. Due to excessive head movements (>3 mm mean displacement), the final analyses include 12 children with ASD (average age: 13.19±2.61, average full-scale IQ: 116±14) and 12 TD children (average age 12.23±2.10, average full-scale IQ: 119±8). The two groups did not differ significantly with respect to age or IQ (See Table 1). All participants were right-handed as confirmed by self and parent reports as well as by examiner observation. Participants were recruited through the Autism Evaluation Clinic at UCLA, and flyers posted throughout the Los Angeles area, and from a pool of subjects who had previously participated in other research studies at UCLA. A prior clinical diagnosis of ASD was confirmed utilizing both the Autism Diagnostic Observation Schedule-Generic (ADOS-G) [33] and the Autism Diagnostic Interview-Revised (ADI-R) [34]. Participants and parents gave written informed consent (assent for children under 13) according to the guidelines of the UCLA Institutional Review Board and were compensated for their participation. All participants were screened to rule out medication use, head trauma, and history of neurological or psychiatric disorders, substance abuse, or other serious medical conditions.

**Table 1:** Participant Characteristics.

Participant Classification	Age	Full-Scale IQ	ADI-R Score	ADOS Score
ASD 1	17.85	134	20	9
ASD 2	15.59	106	45	17
ASD 3	11.84	118	42	8
ASD 4	16.15	127	N/A	N/A
ASD 5	9.78	120	32	12
ASD 6	9.37	93	47	9
ASD 7	11.71	127	43	10
ASD 8	11.24	122	26	10
ASD 9	12.21	121	40	13
ASD 10	13.63	129	38	20
ASD 11	14.77	108	35	14
ASD 12	14.14	91	59	24
TD 1	14.23	134		
TD 2	13.06	119		
TD 3	8.87	116		
TD 4	11.49	115		
TD 5	13.28	104		
TD 6	12.45	119		
TD 7	10.93	128		
TD 8	15.00	125		
TD 9	14.53	115		
TD 10	11.57	116		
TD 11	8.32	118		
TD 12	13.02	124		

### Image acquisition

Images were acquired using a Siemens Allegra 3.0 Tesla MRI scanner. Each child completed 2 functional runs lasting 5 min 8 sec each (152 EPI volumes, gradient-echo, TR = 2000 ms, TE = 25 ms, flip angle = 90°), each with 36 transverse slices, 3mm thick, 1 mm gap, and a 64×64 matrix yielding an in-plane resolution of 3 mm×3 mm. Co-planar high-resolution EPI structural images were acquired to aid image registration (TR = 5000 ms, TE = 33 ms, 128×128 matrix size).

### Stimuli and Task

**Figure 1:**
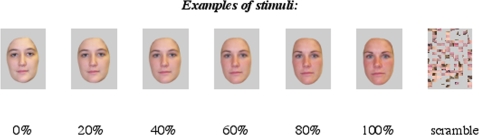
Examples of types of stimuli used in the experiment. For each participant, a series of morphed images were created between the participant's own face and another gender-matched face. This resulted in six face images, from 0% morphing (Self) to 100% morphing (Other).

Stimuli were individually tailored to each child, and consisted of a series of static color images constructed from pictures acquired on a Kodak 3400C digital camera of the child's own face (self) and the face of another person (other). The Other face was chosen from the MacBrain Face Stimulus Set (http://www.macbrain.org/faces/index.htm). We chose faces from this stimulus set for the Other face because subjects had previously viewed faces from this stimulus set in other experiments. Thus, novelty effects were minimized. MorphEditor (SoftKey Corporation, Cambridge, MA) was used to create digital morphs between the subjects' face and the other face (a gender- and race-matched face chosen from the MacBrain Face Stimulus Set), resulting in 6 unique faces, each morphed to a varying extent (0%, 20%, 40%, 60%, 80%, 100%, with 0% being “no morphing of self”). See Figure 1 for examples of types stimuli used in the experiment. The actual stimuli used in the experiment are not shown in order to protect the identity of our participants. Images were edited using Adobe Photoshop to remove external features (hair, ears) and create a uniform gray background. A scrambled control face was created by randomly rearranging one image. The software package Presentation (Neurobehavioral Systems Inc., http://www.neuro-bs.com/) was used to present stimuli and record responses. Stimuli were presented through magnet-compatible goggles (Resonance Technology Inc.) and responses were recorded using two buttons of an fMRI compatible response pad. During each functional run, each of the six morphed faces and the scrambled control were presented 10 times in a random sequence optimized and counterbalanced using the optseq algorithm (http://surfer.nmr.mgh.harvard.edu/optseq/), which provides temporal jitter to increase signal discriminability [35]. Each of the two runs consisted of a different optimized random sequence. Each stimulus was presented for 2 seconds, with at least a 1 second gap between each stimulus presentation. Participants were instructed to press one button with their right hand if the image presented looked like self, and another button if it looked like an other or scrambled face.

### Data Processing and Statistical Analyses

Functional imaging analysis was carried out using FEAT (FMRI Expert Analysis Tool), part of FSL (FMRIB's Software Library, www.fmrib.ox.ac.uk/fsl). After motion correction, images were temporally high-pass filtered with a cutoff period of 50 seconds and smoothed using an 8 mm Gaussian FWHM algorithm in 3 dimensions. The BOLD response was modeled using a separate explanatory variable (EV) for each of the seven stimulus types. For each stimulus type, the presentation design was convolved with a gamma function to produce an expected BOLD response. The temporal derivative of this timecourse was also included in the model for each EV. Data were then fitted to the model using FSL's implementation of the general linear model, with motion components included as confound EVs.

Each participant's statistical data was then warped into a standard space based on the MNI-152 atlas. We used FLIRT (FMRIB's Linear Image Registration Tool) to register the functional data to the atlas space in two stages. Functional images were aligned with the high-resolution co-planar T2-weighted image using a 6 degrees-of-freedom rigid-body warping procedure. The co-planar volume was registered to the standard MNI atlas with a 12 degrees-of-freedom affine transformation.

Statistical analyses were conducted with FSL using mixed-effects models to compute group differences. Higher-level analysis was carried out using FLAME (FMRIB's Local Analysis of Mixed Effects) [36]. Z (Gaussianized T/F) statistic images were thresholded using Z>2.3 and a (corrected) cluster significance threshold of p = 0.05 [37]–[39].

To investigate effects in an *a priori* region of interest (right inferior frontal gyrus, activated in our previous self-recognition study [13]), a mask was derived based on regions activated in the Task-Rest contrast for TD children. Here we use “Rest” to refer to periods between stimulus presentations, during which subjects fixated on a central cross. For this between-group comparison, Z statistic images were thresholded at p = 0.01 (uncorrected voxel p threshold).

Additional ROIs created from anatomical parcellation based on the Harvard-Oxford Structural Atlas provided by FSL [40], [41] were used to query neural response in several regions comprising classical face processing networks [42] and additional regions of particular interest in this study based on our previous work [13], including Brodmann Area (BA) 44, BA 45, middle frontal gyrus, precentral gyrus, angular gyrus, superior parietal cortex, lateral occipital cortex, and fusiform gyrus (also referred to as fusiform face area, FFA).

## Results

### Behavioral

**Figure 2:**
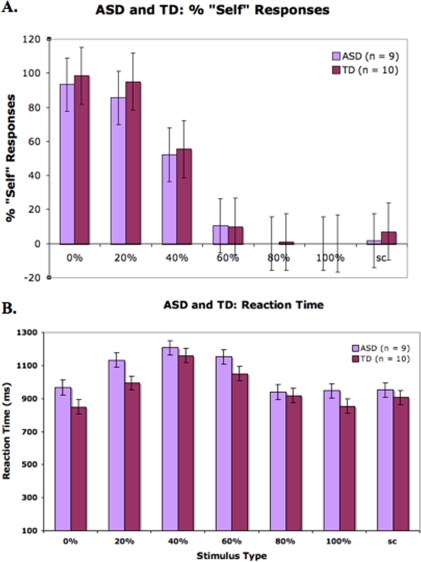
a: Behavioral responses to the task. Both groups of children (ASD and TD) showed decreasing “self” responses as the image presented contained less of the self-face, demonstrating behavioral competence. Error bars represent standard error. b: Reaction Time. There were no significant differences between groups with respect to reaction time.

Figure 2a shows the participants' percent self responses for each of the seven stimulus types, and Figure 2b shows participants' reaction times. Due to technical failure, behavioral responses were not collected from three ASD and two TD children. As expected, the number of self responses diminished as the images presented contained less of the self-face, indicating that both TD and ASD children were able to successfully perform the task. There were no significant group differences in behavioral performance, neither in % self responses nor reaction time.

### fMRI

#### Self>Rest and Other>Rest Contrasts

**Figure 3:**
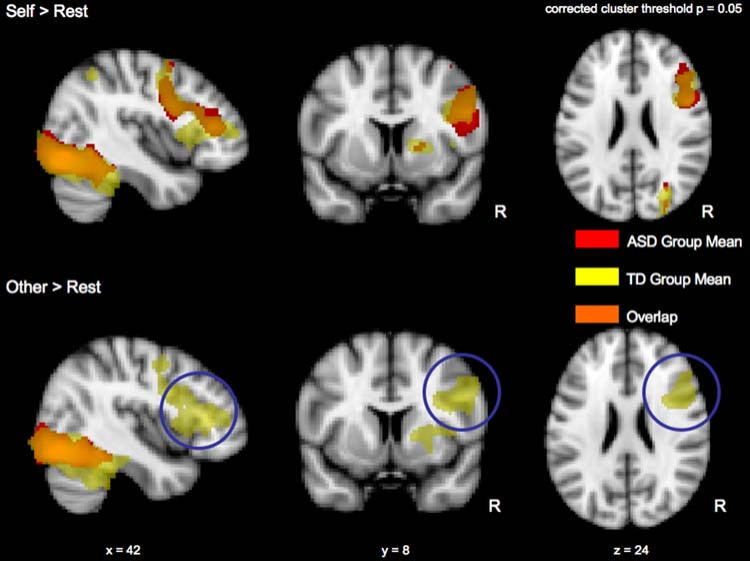
While both groups of children activated the right IFG while viewing of faces of themselves, only TD children also activated this region while viewing faces of others.

The Self>Rest (0%, 20%, and 40%>Rest) contrast for the ASD group revealed activations in the right lateral occipital cortex, right occipital fusiform gyrus, right temporal occipital fusiform cortex, right precentral gyrus, right inferior frontal gyrus, right insular cortex, left lateral occipital cortex, left occipital pole, and left temporal occipital fusiform cortex. The Self>Rest contrast for the TD group revealed a nearly identical pattern of activation (Table 2, Figure 3). Direct comparisons (ASD>TD, TD>ASD) between groups revealed no significant differences for the contrast of Self>Rest in whole-brain analyses (Z>2.3, cluster significance threshold of p = 0.05, corrected).

**Table 2:** Contrasts against resting baseline.

Group/Contrast	Region	MNI Coordinates			Max Z-score
		*x*	*y*	*z*	
**ASD: Self>Rest**	Right Lateral Occipital Cortex	40	−72	−8	6.31
	Right Occipital Fusiform Gyrus	30	−82	−14	5.26
	Right Lateral Occipital Cortex	36	−82	−2	4.68
	Right Temporal Occipital Fusiform Cortex	42	−54	−8	4.4
	Right Temporal Occipital Fusiform Cortex	38	−50	−14	4.2
	Right Temporal Occipital Fusiform Cortex	38	−54	−18	4.17
	Left Lateral Occipital Cortex	−32	−86	−8	5.55
	Left Lateral Occipital Cortex	−40	−82	−12	5.14
	Left Occipital Pole	−14	−100	2	3.92
	Left Occipital Pole	−20	−94	2	3.7
	Left Temporal Occipital Fusiform Cortex	−38	−64	−18	3.67
	Left Temporal Occipital Fusiform Cortex	−34	−48	−20	3.64
	Right Precentral Gyrus	50	6	26	4.61
	Right Frontal Pole/Inferior Frontal Gyrus	40	34	14	4.49
	Right Insular Cortex	34	24	0	3.65
	Right Insular Cortex	32	16	8	3.56
	Right Precentral Gyrus	48	4	40	3.4
	Right Precentral Gyrus	40	0	40	3.19
**TD: Self>Rest**	Right Lateral Occipital Cortex	40	−72	−10	6.89
	Left Occipital Fusiform Gyrus	−30	−86	−14	6.15
	Left Lateral Occipital Cortex	−42	−82	−12	6.06
	Right Lateral Occipital Cortex	46	−62	−16	5.83
	Right Lateral Occipital Cortex	28	−76	26	5.1
	Right Temporal Occipital Fusiform Cortex	38	−42	−22	4.8
	Right Precentral Gyrus	50	6	26	4.59
	Right Frontal Pole/Inferior Frontal Gyrus	40	34	12	4.23
	Right Insular Cortex	32	18	−8	4.21
	Right Frontal Pole	38	48	10	3.85
	Right Frontal Pole	42	48	10	3.84
	Right Frontal Operculum/Frontal Orbital Cortex	34	28	4	3.77
**ASD: Other>Rest**	Right Occipital Fusiform Gyrus	28	−84	−16	6.09
	Left Occipital Fusiform Gyrus	−30	−84	−10	5.06
	Right Lateral Occipital Cortex	42	−76	−14	4.78
	Left Temporal Occipital Fusiform Cortex	−32	−48	−22	4.52
	Right Lateral Occipital Cortex	40	−74	−8	4.51
	Left Occipital Fusiform Gyrus	−18	−88	−14	4.31
**TD: Other>Rest**	Right Occipital Fusiform Gyrus	30	−84	−16	7.02
	Right Lateral Occipital Cortex	42	−72	−14	6.15
	Left Occipital Fusiform Gyrus	−30	−84	−16	6.12
	Left Occipital Fusiform Gyrus	−42	−66	−24	6.07
	Right Occipital Fusiform Gyrus	22	−88	−12	6.04
	Left Temporal Occipital Fusiform Cortex	−36	−58	−18	5.88
	Right Inferior Frontal Gyrus	52	14	30	4.61
	Right Precentral Gyrus	48	6	28	4.55
	Right Inferior Frontal Gyrus	42	32	10	4.29
	Right Frontal Operculum/Insular Cortex	32	26	6	3.71
	Right Frontal Operculum Cortex	40	12	6	3.32
	Right Insular Cortex	30	16	2	3.22

**Figure 4:**
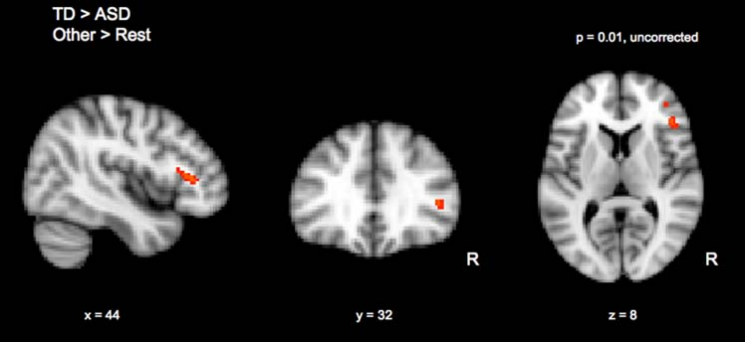
TD children showed greater activation to other faces than children with ASD, specifically in the right IFG region-of-interest.

The Other>Rest (60%, 80%, or 100%>Rest) contrast for the ASD group showed activations in right occipital fusiform gyrus, right lateral occipital cortex, left occipital fusiform gyrus, and left temporal occipital fusiform cortex. The Other>Rest contrast for the TD group showed activations in right occipital fusiform gyrus, right lateral occipital cortex, right inferior frontal gyrus, right precentral gyrus, right insular cortex, right frontal operculum cortex, left occipital fusiform gyrus, and left temporal occipital fusiform cortex. While direct statistical comparisons between groups (ASD>TD, TD>ASD) revealed no significant differences for the contrast of Other>Rest in whole-brain, cluster corrected analyses (Z>2.3, cluster significance threshold of p = 0.05, corrected), the within-group results for the Other>Rest contrast revealed striking differences between TD and ASD children in the right prefrontal cortex. Specifically, while TD children activated the right inferior frontal gyrus (rIFG) while viewing images of others, there was no change in activity between Other versus Rest for the ASD children (Table 2, Figure 3). For this reason, and based on previous studies implicating the rIFG in similar face-processing tasks [13], [43]–[45], we conducted a subsequent between-group analysis focusing specifically on the right prefrontal cluster, using a functionally defined region-of-interest (ROI) derived from the group mean of the TD children in the Task-Rest contrast. As shown in Figure 4, the results of this analysis showed greater rIFG signal change for Other>Rest in TD children than in ASD children.

#### ROI Analyses

**Figure 5:**
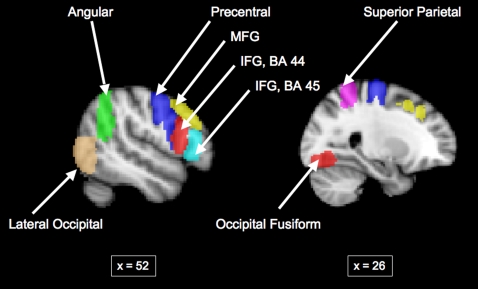
ROIs based on Harvard-Oxford atlas used to probe differences between TD and ASD groups.

**Figure 6:**
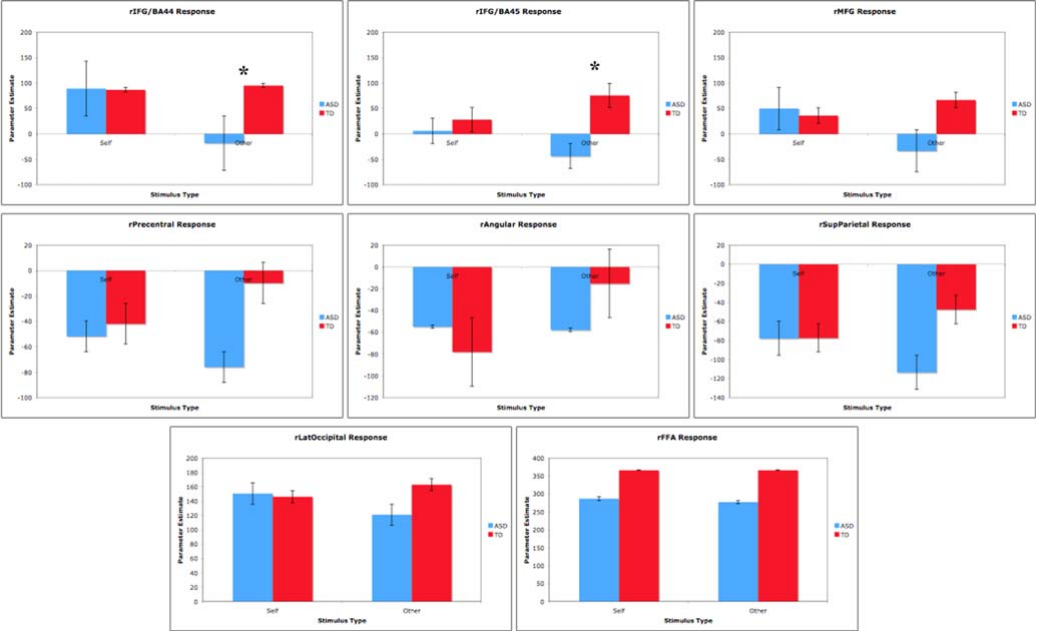
ROI analyses indicated hypoactivation to Other faces in the ASD group in BA 44 and BA 45.

Using anatomically defined ROIs provided by the Harvard-Oxford Structural Atlas, signal intensities were estimated within the following brain regions in the right hemisphere: BA 44, BA 45, middle frontal gyrus, precentral gyrus, angular gyrus, superior parietal cortex, lateral occipital cortex, and fusiform gyrus (Figure 5). For all of these regions, no significant differences were found between the ASD and TD group during viewing of Self faces (Figure 6). Additionally, the only regions in which group differences in signal intensity for viewing Other faces approached significance were BA 44 (t(22) = 1.717, p = 0.07) and BA 45 (t(22) = 1.717, p = 0.08), confirming the results of our between-group comparison based on the functionally defined ROI in this area.

## Discussion

The representation of self and other may be fundamentally altered in autism spectrum disorders [4]. While previous work has largely emphasized deficits in interpersonal interaction in ASD, current empirical work emphasizes the need for understanding differences with respect to both *interpersonal* (social) and *intrapersonal* (self-referential) cognition [10]. Previously we and others have shown that in normal adults, self-recognition is accompanied by signal changes in a right hemisphere network including the inferior frontal gyrus and inferior parietal lobule [13], [43], [44], consistent with earlier work implicating preferential right hemisphere involvement in self-recognition [46]. Here we used this paradigm to test whether children with ASD recruit the same brain areas during self- and other-face processing as do TD children. We showed that while self- and other-face processing involve virtually overlapping right frontal activity in TD children, children with ASD only exhibit such activation when viewing their own faces. ROI analyses confirm that ASD and TD children differentially activate the rIFG (specifically BA44 and BA45) during viewing of other faces. Specifically, children with ASD activate the rIFG less than do TD children during viewing of other faces. In every other ROI within the broader face-processing networks we examined, we observed no significant group differences during viewing of self or other faces. Thus, consistent with clinical observations of higher levels of self-focused behavior in autism, these children show decreased neural response to viewing faces of others compared to viewing faces of themselves. While hypoactivation of the fusiform gyrus to faces has been previously reported [47], this region does not appear to be involved in self-other distinction as examined in the current study. One can speculate that normal engagement of several brain regions by self faces and reduced activity to other faces in the rIFG might be related to the social impairments characteristic of children with ASD.

Previous work on the neural basis of self-face recognition in adults suggests that while the right inferior frontal gyrus responds most strongly to self-faces, it also shows signal increases (although weaker) above baseline to personally familiar faces [13], [45]. The fact that the TD children examined in this study do not yet show self-other differentiation in the right IFG suggests that our cross-sectional design captures a period in cognitive development where this distinction is not robustly represented, and a great deal of self-other overlap exists. One may speculate that as the representation of self becomes more differentiated in post-adolescent development, the rIFG activation to self relative to others becomes more selective. In contrast, children with ASD appear to not exhibit this neural overlap. As our study did not use personally familiar faces as controls, we cannot address the interesting question of whether the response of the rIFG of children with ASD is modulated by personal familiarity, as has been suggested for the fusiform face area [48].

Our paradigm required participants to explicitly evaluate facial identity to decide whether the image presented resembled themselves. Both groups of children behaviorally demonstrated a diminishing “self” response as the image presented contained a smaller percentage of the self face, indicating successful self-other discrimination. This result is consistent with behavioral work demonstrating successful visual self-recognition in most children with ASD [28], [29], [49]. Our previous work has implicated the right inferior parietal lobule in this type of discrimination, as virtual lesions induced with rTMS reduced subjects sensitivity to detecting self faces [14]. The right IPL is thought to be part of a circuit mediating complex own-body perception [50], and contributes to the sense of agency, or the feeling of generation of action [51]. We saw no significant group differences during viewing of self-faces in this region, suggesting that the perception of the self face as part of one's own body is not altered in children with ASD. Rather, it appears that the mechanisms for detecting self-other similarity, likely dependent on the right inferior frontal gyrus, are dysfunctional in these children. The mechanisms implemented by the rIFG likely mediate differences between ASD and TD children in social interactions that are more complex than those tapped by our self-other discrimination task.

Social cognition researchers have previously suggested that understanding of others' experiences may involve the activation of shared affective neural networks that enable us to “feel the emotions of others as if they were our own” [52]. There is now a fairly substantial literature documenting the existence of shared neural representations that bridge the gap between the self and other in various domains, including the experience of pain [53], touch [54], and emotion [55], [56]. Such “embodied simulation” accounts of action and emotion understanding go by many names (e.g. “shared manifold of intersubjectivity” or “intentional attunement” [57], “shared representations” [12], “shared circuits” [58]), but they all support the notion that one of the ways by which an individual makes sense of the social world is by using the same brain systems that are used for self-related experiences in order to understand others. This overlap in self- and other-representation may breakdown in autism, which is characterized by decreased empathy [59] and theory of mind impairments [8]. The restricted social interests of individuals with autism may thus reflect a fundamental lack of appreciation of self-other similarities, which may be the result of altered mirroring mechanisms in the brains of such individuals. There is also mounting evidence for dysfunction of the so-called mirror neuron system, which is implicated in social cognition [60], in individuals with autism [18], [20]–[25], [61].

One possible explanation for the lack of rIFG response to others observed in this group of children with ASD may be the fact that from a young age autistic individuals seem to lack the motivation for orienting to social cues that their typically developing counterparts demonstrate [62]. Indeed, some have theorized that the lack of interest in social stimuli evident in autistic children may result from the fact that ASD children do not find these stimuli to be rewarding [63]. Previous reports have shown that when children with ASD are instructed to pay attention to specific aspects of social stimuli (e.g. a person's facial expression or tone of voice), neural responses more closely resemble those of TD children [64], [65]. Thus, while socially relevant stimuli such as other's faces may not automatically engage the attention of children with ASD who are less driven by social motivation, explicit instruction to attend can ameliorate this effect. In the present study, however, the task instructions focused the children's attention to detecting the self, perhaps further decreasing the amount of attention directed toward others. Future work should examine whether simply modifying task instruction (i.e. asking participants to detect the presence of other faces in the morphs) may “normalize” neural activity to other faces in children with ASD.

In conclusion, we find that children with ASD do not activate shared regions for self- and other-face processing, as do TD children. Reduction of activity in right prefrontal areas during other-face processing may be a neural signature of reduced social engagement and understanding in these individuals.
